# The East Africa Trachoma/NTD Cross-border Partnership

**Published:** 2015

**Authors:** Teshome Gebre, Girija Sankar

**Affiliations:** Regional Director for Africa: International Trachoma Initiative, Addis Ababa, Ethiopia.; Senior Programme Associate: International Trachoma Initiative, Atlanta, USA.

**Figure:**
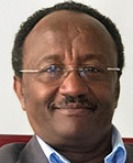
Teshome Gebre

**Figure:**
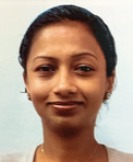
Girija Sankar

Eastern Africa, which comprises Eritrea Ethiopia, Kenya, South Sudan, Sudan, Tanzania and Uganda, has the highest burden of trachoma in the world. In sub-Saharan Africa, there are 1,274 districts known to be endemic for trachoma (i.e., the incidence of trachomatous inflammation – follicular (TF) is > 5%). Of these, 769 are found within the seven Eastern African countries, which represents over 60% of the trachoma endemic districts and 50% of the at-risk population in sub-Saharan Africa.

**Figure:**
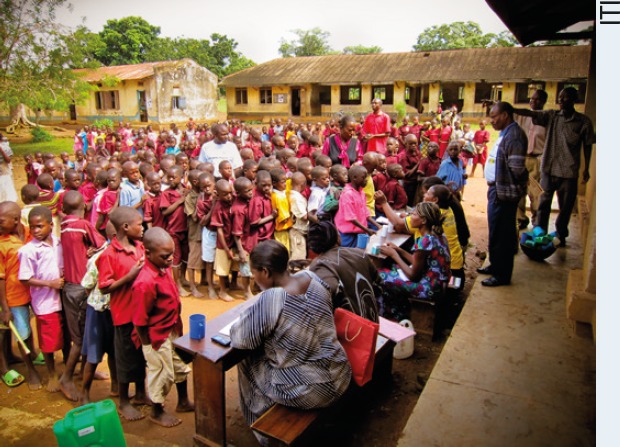
School children line up to receive Zithromax^®^. UGANDA

Cross-border movement amongst the pastoralist communities along the long, porous, common borders within Eastern Africa are highly significant in terms of disease transmission and control. It is likely that no single East African country can achieve and sustain elimination unless its neighbours do the same.

As the target date of the year 2020 for the Global Elimination of Blinding Trachoma (GET 2020) approaches, it is evident that we need to coordinate, intensify and align our efforts within each endemic country and across the common borders. We need a common platform to discuss funding, logistics and drug supply chain management issues. Most importantly, we need to share best practices and draw lessons from the challenges facing each individual country programme.

In order to achieve this, a sub-regional consultative meeting involving the seven countries in East Africa was held to deliberate on these issues and decide future directions. This is similar to a cross-border initiative established in West Africa in 2010 to address neglected tropical diseases in five countries. The initiative, called ‘END in Africa’, is supported by USAID and managed by an NGO called FHI360.

The first Eastern Africa regional meeting took place in July 2015 in Machakos, Kenya with the theme ‘Strengthening Cross- border Collaborations and Partnerships to achieve Trachoma/NTD Elimination.’ The seven countries in the region were represented by two Ministry of Health delegates each: the national NTD coordinator and the national trachoma programme focal person. Nearly all of the relevant NGOs and funding partners in the region were in attendance, and it was decided to form a coalition, known as the East Africa Trachoma/NTD Cross-border Partnership. Overall, participation in both plenary and group discussions was strong and enthusiastic. The future of the partnership, and its impact on trachoma/NTD elimination, shows promise.

The partnership aims to further the global goal of trachoma and NTD elimination by enhancing programme performance and instilling a spirit of urgency and healthy competition among the Eastern African countries. The initiative's specific objectives can be summarised as follows.

1. Create a regional platform for exchanging experiences among participating countries and their respective partners.
2. Identify programmatic bottlenecks and challenges and recommend practical solutions.
3. Ensure that countries are adhering to World Health Organization (WHO)-recommended strategies and standard operating procedures and guidelines.
4. Assess resource gaps for SAFE implementation (Surgery, Antibiotics, Facial cleanliness and Environmental Improvement) and advocate for resource mobilisation.
5. Design strategies for tackling cross-border challenges.

**Figure:**
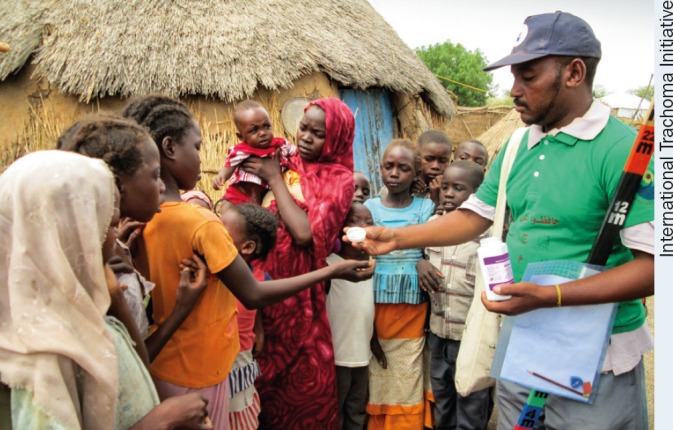
Mass drug administration in a community. SUDAN

**Figure:**
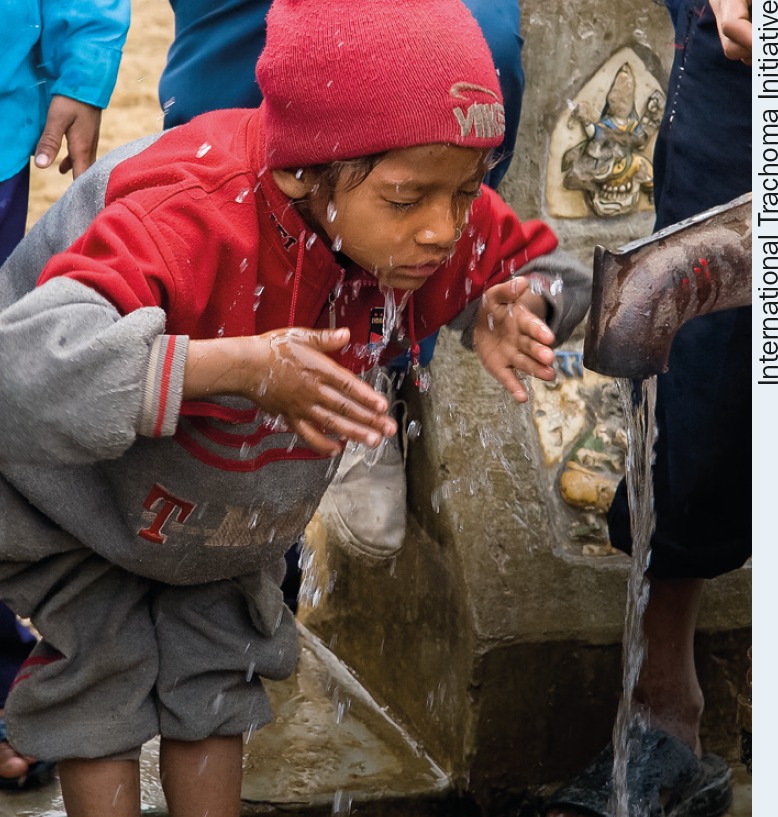
Face washing. ETHIOPIA

Several country-specific and general recommendations were outlined at the conclusion of the meeting. The partnership recommended that:

- The International Trachoma Initiative (ITI) acts as the secretariat for the group and that it organises an annual meeting to discuss East African cross-border issues.
- The secretariat set up a communication platform for information sharing between partners.
- ITI propose clear guidance for countries seeking Zithromax^®^ donations for treatment in camps for internally displaced persons and refugees.
- Partners look for opportunities to promote awareness of cross-border trachoma collaborations and conduct activities for advocacy and awareness-raising.
- Each country's Ministry of Health advocate for government and private funding to support cross-border issues and that they match and leverage partner funding.
- All partners engage with WASH (water, sanitation and hygiene) sector stakeholders in implementing the full SAFE strategy.
- The secretariat work with country NTD point persons to follow upon recommendations.

Eastern African countries should consider regional trachoma elimination plans that allow for joint planning and coordination of antibiotic distribution along the endemic border areas to reduce possible reinfection and accelerate towards elimination goals. Collaborative programming allows neighbouring countries to identify common strategies for trachoma control in refugees and internally displaced persons. Opportunities for collaborative trachoma control are further enhanced when countries with common borders are supported by the sa me funding and implementation partners. A follow-up virtual meeting has taken place in early 2016. The next in-person meetingwill be held in Arusha, Tanzania.

